# Mother-to-child transmission of human immunodeficiency virus (HIV) – experience of two AIDS centers from Romania

**DOI:** 10.1186/1471-2334-14-S4-P11

**Published:** 2014-05-29

**Authors:** Liana Precupescu, Corina Itu, Carmen Chiriac, Erzsébet Iringó Zaharia-Kézdi, Mihaela Lupşe

**Affiliations:** 1 Iuliu Hațieganu University of Medicine and Pharmacy, Cluj-Napoca, Romania; 2 University of Medicine and Pharmacy, Tîrgu Mureş, Romania

## 

Mother-to-child transmission of human immunodeficiency virus (HIV) infection decreased in the last years due to the implementation of: HIV testing, antiretroviral (ARV) medications, delivery by cesarean section and discouraging breastfeeding. In Romania, pregnant women could acquire HIV infection as adults or could have a long history of disease and ARV treatment (ART), belonging at the Romanian cohort.

Aim: to evaluate the risk factors for HIV transmission from mother-to-child during pregnancy and delivery.

Retrospective study based on patients’ electronic medical records files and AIDS database (Cluj and Mureş AIDS Center) of all children born by HIV infected women during Jan 2008 to Dec 2013 for risk factors of HIV transmission. We evaluated women for related risk factors: age, AIDS stage, Romanian cohort membership, plasma viral load during pregnancy, HAART treatment during pregnancy, mode of delivery, infant ART prophylaxis. Statistical analysis was performed with chi square test and multiple regression.

54 newborns were born by 45 HIV infected women during the study period, 8 (14.81%) of them being diagnosed with HIV infection. The women’s average age was 23.82 (min 18, max 38), 29 women belonged to the Romanian cohort. The AIDS stage was: A for 12 patients, B for 19 patients and C for 23 patients. 6 out of 45 women (13.33%) didn’t receive ART during pregnancy, being diagnosed during labor. Plasma viral load during pregnancy was determined for 36 women: undetectable for 8 women, less than 400 copies/mL for 5 women and more than 400 copies/mL for 23 patients. Delivery was with elective caesarean section for 47 newborns (87.03%). Infant ART prophylaxis was administered in 48 newborns (88.88%). The risk of mother-to-child HIV transmission was significantly lower with: ART during pregnancy (p<0.001) not important of ARV association, cesarean section (p<0.001) and infants’ ART prophylaxis (p=0.03).

The risk of mother-to child HIV infection is high in Romania; the percent of women not tested for HIV infection during pregnancy is low; the risk of HIV infection transmission was significantly lowered by ART during pregnancy and in infant, as prophylaxis, and by elective caesarean section.

